# Paretic Dementia and Life Insurance*Read before the New York Society of Medical Jurisprudence.

**Published:** 1884-06

**Authors:** James G. Kiernan

**Affiliations:** Chicago


### ARTICLE II.

**PARETIC DEMENTIA AND LIFE INSURANCE.*** A Study of the Dwight Case. By JAMES G. KIERNAN, M.D., Chicago.

The Dwight Case was a decidedly complex one, and what in my opinion, was the most important of its problems, was not at all considered in the decision which terminated it. Whether Col. Dwight did or did not commit suicide to enrich his family, seems to me to be a matter of subordinate importance. The reasoning adopted to prove that suicide is a sometimes sane act, was not at all suited to its purpose, and from a scientific standpoint, worthless. No alienist worthy of the name will claim that suicide *per se* is an evidence of insanity. Suicide in a con-

*Read before the New York Society of Medical Jurisprudence.

scientious Roman Catholic would be presumptive evidence of insanity; in a free-thinker it would be presumptive evidence of nothing. The individual is the proper means of comparison, not different races, as proposed by some solicitors of life insurance companies. Any act which is in full consonance with an individual's circumstances and education and surroundings, cannot *per se* be regarded as an insane or sane act; it has a negative value only. The issue of suicide raised in the Dwight case, and made the main issue, led to a waste of time and glorified "experts" who were hitherto unknown. As gleaned from very conflicting evidence, the facts of the case are as follows: Col. Dwight, a man of prudent habits, suddenly became addicted to extravagant speculations, evidently engendered by the existence of stupid delusions of grandeur. He, at the same time, presented evidence of organic brain disease. These speculations had almost reduced him to bankruptcy, when he suddenly insured his life for $300,000 in various life insurance companies, dying, after one premium had been paid, his friends claimed, from the effects of a congestive chill conjoined with an overdose of morphine. The companies claimed that the Colonel committed suicide by strangulation. On autopsy, old and recent pachymeningitis with adhesions to the surface of the brain were found. The abdominal organs presented evidences of having been the seat of some malarial disease. There was noticed on the neck a peculiar mark which resembled that of a rope, but might have been produced by a tight necktie. To the alienist, the history above given points to the existence of paretic dementia at a period prior to the time the Colonel first entered into these wild speculations. Under such circumstances the question naturally arises, whether policies of insurance would have been secured by a paretic dement.

What is paretic dementia? It is a very well marked mental affection, easily diagnosed by a trained observer, but readily escaping the notice of laymen and the average untrained physician.

Two ex-commission merchants entered into a partnership involving several million dollars. A gambler won millions from his fellow-patients, and based his delusions on theirs. One patient, if corrected about his delusions, would call on a mysterious double for confirmation, and then answer in the capacity of this double himself. The patient, while given to extravagant ideas of wealth, may be able to give true accounts of his pecuniary circumstances if kept to the point by leading questions.

The paretic dement is very apt to accept plans and schemes which look alluring; he is only able to see it from this standpoint, and everything which tends in any way to exalt his importance is seized upon by him. An apt illustration, and one which bears upon the subject under discussion, is the following case reported by Legrand du Saulle.* A physician well known in science, had had for more than nine years an insurance of $20,000 on his life. He suddenly gave unequivocal evidences of mental impairment, went and came rapidly, and wrote and spoke a great deal. He had an exaggerated opinion of his labors, vaunted his success in practice, and boasted of his professional ability. In one of his walks he accidentally met the president of the company in which his life was insured. After a long conversation, Dr. Blank said that his life was assured for an insignificant sum, and that he had resolved to increase the amount to $100,000. He was referred to the proper authorities, who consented. The policy was prepared, and all that was needed to complete the assurance was a receipt for the same. The agent, who was ready to deliver the policy on the signature of the receipt, suddenly noticed, just as the doctor was about to sign this, that the latter talked rather freely and loudly. Suspecting intoxication, he put off the delivery of the policy until Dr. Blank should become sober, claiming to have forgotten some important formality. The following day the physician was sent to an hospital for the insane, where he died in six months from paretic dementia. The company paid the $20,000 to his widow, and esteemed itself happy that it did not have to pay the $100,000 for which the husband had desired, in a fit of pathological temerity, to assure his life.

The appetite, as a rule, is good, even excellent, and the sudden appeasement of a seemingly good appetite in lunatics is sometimes, as has been shown by Dr. Workman, of value as a diag-

---

* *Gazette des Hopitaux*, Sept. 23, 1883.

nostic hint. The remissions are of interest from the medico-legal and diagnostic standpoint. They furnish one of the numerous stumbling blocks for the dilettante alienists who infest our courts. Sauze describes them as of three kinds: Those in which there is remission of the mental and physical symptoms simultaneously; those in which there is remission of the mental symptoms alone; finally, those in which there is remission of the motor symptoms alone. Mickle found the first two types common, but the third he had never observed. As has been pointed out by Baillays, there is, as a rule, an amelioration of the mental symptoms, the motor symptoms persisting. This has also been the experience of Bayle, Ferrus and Rodriguez. Lubimoff and Doutrebente have claimed that the remissions are sometimes complete restorations to health. Spitzka, Westphal, Mickle and myself have seen but very rarely such complete remissions. Doutrebente and myself have found the speech trouble least affected by the remission. Lunier has reported a case in which seven remissions occurred. The case was of twenty-seven years' duration. The disease has a very bad prognosis, both as to life and recovery, as it causes death in, as a rule, from three to four years. The gross lesions usually found on autopsy are those already described in the Dwight case.

Enough has already been detailed to illustrate the predominant characteristics of this disease. It will be obvious that the intellectual powers of a paretic dement are such as would make him an easy victim of the blandishments of any "sharp" insurance agent who would believe him, from his apparently excellent health, to be a good risk. Once started in the life insurance direction, the paretic dement would keep on, like Legrand du Saulle's patient, increasing the insurance on the life of the valuable citizen he believes himself to be, since the paretic has a tendency to adopt and extend ideas derived from others, as is shown by the case cited by Spitzka, of the paretic dement who, saved by his wife from a paretic suicide, proposed to erect a monument to her for saving the life of so valuable a citizen. It is curious how often this psychosis escapes observation by the non-alienist physician and laymen, when compared with the ease with which it is diagnosed by even asylum attendants familiar with it. The two cases

(already cited) as occurring in New York State were two cashiers of banks who had been paretic dements for at least two years, and had nearly brought the bank to a condition of insolvency by simple lack of attention to business; their mental condition had not been at all suspected by their business associates. In Chicago, a saloon-keeper with a paretic delusion of having inherited $2,000,000, was interviewed by the reporters of every paper in the city, by none of whom was his mental condition suspected until his friends took measures to have him placed under treatment in the County Hospital for the Insane. There is, therefore, nothing antagonistic to the existence of paretic dementia in Col. Dwight, in the fact that he insured his life for the amount stated, and that his mental condition escaped observation from the business men of the companies. It may, however, be urged that the medical officers of the companies would detect the psychosis. He who knows how these medical officers are chosen—either from relatives, connections of officials of the companies, or by agents,* who tell the physician that "he must not be too strict;" or for political reasons to influence State legislatures, from medical politicians* who indulge in "graveyard insurance," in which they get caught sometimes, but not as a rule, will scoff at the idea that men like these, who neither know nor care to learn anything of psychiatria, could be of value in the detection of insanity. Even in France, where the physicians are supposed to be better trained than the graduates of our diploma factories, the one case cited shows the value of non-alienist medical examination, and the same point is still more strongly illustrated by another case cited by Legrand du Saulle: Two brothers presented themselves at the office of a French alienist. The elder entered the office first, and asked the alienist to examine with care the patient brought him. "There seems to be nothing the matter with my brother," said he, "but he is no longer the same." The physician, after a long examination, said, "Your brother is in the initial stages of paretic dementia." Explanations were then given, and the prophecy made that the patient would die in three or four years. The following day an assurance of $20,000 was

* Actual occurrences in Chicago.

placed on the life of the patient. Three years after, the brother pocketed this amount.

The first stage is usually ushered in by emotional excitement, generally of an expansive type. The patient is hilarious, perhaps even boisterous. He is likely to make extravagant purchases or lavish gifts. His memory is not unlikely to be capriciously defective. Carelessness in attire may suddenly present itself. On the other hand, the patient may suddenly become fastidious about the use of flashy attire. A patient in Chicago displayed this at his trial for insanity to such an extent as to be called the insane "dude."

The patient, as in cases observed by Hammond, Lélut, Baillarger, Parot, Billod, Brierre de Boismont, A. Sauze, Maudsley, Burman, Fabre, Darde, Mickle, and Voisin, may commit theft in a stupid manner. I have observed cases in which theft was the first obvious evidence of the patient's insanity, which was not recognized until he had been tried and condemned to the penitentiary. In a case which recently occurred in New York, a man who was first arrested for stealing flowers in open day and in presence of a large crowd, was upon examination found to be suffering from the initial stage of paretic dementia. Le Grand du Saulle has been called on to determine the mental status of many paretic dements the first observed symptom of whose disease was theft. If the patient be an officer in monetary trust, errors in his accounts leading to suspicions of embezzlement may occur. In most cases, as in two recently reported from New York, this is due to forgetfulness. Le Grand du Saulle reports the case of a cashier who suddenly became addicted to visiting places of ill-repute, neglected his accounts and embezzled money, displaying soon after evidences of the third stage of the disease.

Eroticism, varying from tendencies to form improper marriages up to the wildest lasciviousness, as in cases reported by Guislain, may make its appearance. Moreau (de Tours) says that the sentiment of love in its ideal sense is very often the first symptom of this affection; the genital excitement appearing later. The first tendency is often noticed in hitherto fanatically orthodox Jews who suddenly contract marriages with Christians. In a case cited by Le Grand du Saulle, a man suddenly married a prostitute and legitimized, by acknowledgment according to the French law, her two children, dying soon after in an asylum. This sexual excitement may manifest itself in indecent exposure of person. I have observed the case of a hitherto respectable physician who suddenly indecently exposed his person; fined for this he immediately exposed it on leaving the court-room, and the fine was doubled, but he again repeated the offense. In consequence of this he was committed to prison, and on his release came to Chicago, where he was found to be a paretic dement, dying in the Cook County Hospital for the Insane.

Wild speculations may be suddenly entered into. In a case cited by Sankey, a paretic dement whose mental condition had been hitherto unsuspected suddenly rose in a meeting of stockholders of a concern on the point of bankruptcy and offered to purchase all the stock. In a case cited by Spitzka, a hair-dresser bought up all he could find of a certain shade of gray hair. The delusions which may occur at this time, as a rule, are expansive in character. The patient is immensely wealthy, rich or strong. One of my patients, who was a little less than five feet high, claimed an altitude of ten thousand feet and a corresponding breadth. He was placed next a paretic slightly taller, and on being asked if the latter was not taller than he, responded that he was, but replied in answer to the question how tall he (the first patient) was, "ten thousand feet." The patient, who claims to be King Brian Boru, will answer to the sobriquet of Michael Mahoney, and give the latter as his name in answer to a question to that effect. One of my patients who had been a merchant made a list of his property consisting of patents, among which was a receipt for clam chowder, this he valued at a billion, and the other thirty-three patents for watches and railroads at one hundred millions. One of Hammond's patients owned all the trout streams in the country. The patient confuses the day dream with the actual. The delusions shift and vary. The man who is worth $1,000,000 to-day is worth fifty in ten minutes more. If contradicted, his allusions become exaggerated. The paretic dements, unlike the majority of the other insane, do not recognize the insanity of their co-invalids. In fact, one paretic dement often accepts another's delusions as a basis for his own. One paretic dement in the New York City Asylum for the insane was going to secure an immense increase of capital by marrying his daughter to a fellow paretic dement.

In Germany a medical officer of a railway corporation stated the symptoms of paretic dementia, presented by a 43 year old man, were only changes due to old age.

Hanot* in a recent paper calls attention to the dangers life insurance companies are exposed to from this class of patients, who, as already stated, escape the diagnostic ability of the average insurance company physician, and it will be obvious that the average untrained physician is worthless in the detection of this psychosis. With all these facts before him, the alienist will not hesitate to say that Col. Dwight was a paretic dement when he effected the insurance on his life. Whether he committed suicide or not, is foreign to the real question in the case. Is an assurance effected on the life of the paretic dement by himself or his relatives, in good faith, binding on the company making such assurance? The Supreme Court of Iowa* has decided that persons of unsound mind are to be held bound by an executed contract or conveyance when the transaction is fair and reasonable, and in the ordinary course of business, and where the mental condition of the party is unknown to the second part, and the parties cannot be placed in *statu quo*. The converse of this necessarily follows: A paretic dement cannot be guilty of fraud, for fraud implies a knowledge of the true state of things, and of this knowledge the paretic dement is destitute. Col. Dwight effected an assurance on his life because he was a paretic dement. If contracts with the insane under the circumstances mentioned by the Iowa decision are binding, then his policies were binding on the companies. If the policies were binding on the companies, no suicide by him could vitiate them. It may be admitted that even assuming the truth of all the circumstances, it was an injustice to burden the companies with the results of the actions of an insane man. With the consequences of a law a judge has nothing to do; his duty is to declare what that law is, and the same holds good of a scientist and scientific truth.

---

**Northwestern Reporter*, June 18, 1881.

*Annales d, Hygiene, 1883.

The companies were themselves to blame for accepting such a risk, a risk offered by the would-be policy holder in good faith. It may seem hard on the companies, but it would also be hard on the family of other paretic dements if they should, in consequence of the precedent set in this case, be deprived of the provision made for them. The life insurance companies use all means to detect fraud and to ascertain the value of risks. If such means prove insufficient, not through fraud of the policy holder, but through the incapacity of the companies' agents, the company should be held responsible. For these reasons, the present writer believes that the decision in the Dwight case was fully warranted, even supposing the deceased committed suicide. Another aspect of the case presents itself: Supposing Col. Dwight, a paretic dement, insured his life and then, with a paretic idea of smartness, committed suicide to compel the company to suffer for some imagined affront, or with the vague idea of making money for his friends, would his act vitiate his policy? Under the Iowa decision the writer believes not, but would not like to express an opinion of the abstract justice of such a decision; legally he believes the policy could not be declared void; however seemingly unjust its non-voidance, or rather the full requirements of all its provisions under the circumstances would be. The question simply resolves itself into this: Can a contract, which, *per se*, involves an element of risk, all means of diminishing which risk are supposed to be taken by one of the persons making the contract, be regarded as void because, without fraud on the part of the other person making the contract, the risk proves greater than was anticipated. Life assurance contracts are based on the theory that the company ascertain the exact condition as regar ds disease. If the assured have an ordinary physical disease, unknown to himself and the examiner, and die from such disease, the company is held bound to pay the amount of the policy. It would seem that the same rule should hold good in the case of a paretic dement. The present paper has been written simply to raise this question for discussion. The writer believes that the policy and stockholders suffered unjustly in the Dwight case from the " inventive stupidity of agents and medical officers, and from the legal advisers making a fight on the question of fraud, where one should have been made on the question of contracts with the insane."

